# Montana

**Published:** 1897-08

**Authors:** 


*Montana.* This State is to-day one of the most aggressive in the creation and maintenance of a veterinary sanitary police system of any of the United States. With an energetic and broad-gauged State veterinarian, there is more being done that will prove of inestimable value to this State and greatly encourage similar plans in other States. Movements of all live-stock are accompanied by health and examination certificates from the State or deputy State veterinarians. Quarantine regulations are rigorously enforced when deemed necessary. A proclamation of the Governor on March 13th announces the conditions under which cattle may enter the State from below the quarantine line of Texas-fever. A similar proclamation relative to the entering of sheep within the State, compelling a thorough examination by the State veterinarian or a District sheep inspector, establishing quarantine regulations necessary for the control and eradication of scab in sheep. Another order proclaiming the general prevalence of tuberculosis and fixing the requirements necessary for entrance of other cattle in the State. Also as to swine, and the various penalties are enumerated applying to those who violate these sanitary measures in exposing, shipping, or driving over roads any animals affected with any contagious or infectious diseases. Another proclamation as to infectious mange in horses and mules, limited in this instance to a well-defined locality, and quarantine measures necessary for its control.

**Treasurer Robertson still yearns for the golden shores of California.**

The State Board of Stock Commissioners have issued in concise form all the former laws now in force relative to live-stock and agriculture. It contains all the prescribed duties of the commission and the duties and powers of the State veterinarian.

The official deputy veterinarians of Montana so far appointed are as follows: Dr. T. D. McGregor, Butler; H. H. Nelson, Cascade; Charles Brown, Miles City; Dr. J. E. Wamsley, Choteau; H. C. Pickett, Helena; Dr. E. V. Wilcox, Bozeman; Dr. Foster, Fort Assinaboine; and T. F. Bryson, Merrill.

North Dakota officials will recognize all health certificates of the Montana State and deputy veterinarians.

**Look out for the report of the Publication Committee.**

Deputy veterinarians in Montana are paid five dollars per day and necessary expeness.

Sarcoptic mange has been existing in one county of Montana for several years, although measures have now been inaugurated to stamp it out.

